# Conversion of human adipose-derived stem cells into functional and expandable endothelial-like cells for cell-based therapies

**DOI:** 10.1186/s13287-018-1088-6

**Published:** 2018-12-17

**Authors:** Fuyi Cheng, Yujing Zhang, Yuan Wang, Qingyuan Jiang, Cheng jian Zhao, Jie Deng, Xiaolei Chen, Yunqi Yao, Zhemin Xia, Lin Cheng, Lei Dai, Gang Shi, Yang Yang, Shuang Zhang, Dechao Yu, Yuquan Wei, Hongxin Deng

**Affiliations:** 1 State Key Laboratory of Biotherapy and Cancer Center/Collaborative Innovation Center for Biotherapy, West China Hospital, Sichuan University, Ke-yuan Road 4, No. 1, Gao-peng Street, Chengdu, 610041 Sichuan People’s Republic of China; 2 Department of Obstetrics, Sichuan Provincial Hospital for Women and Children, Chengdu, People’s Republic of China

**Keywords:** Human adipose-derived stem cells (hADSCs), ETV2, EC-like cells, Angiogenesis, Regenerative medicine

## Abstract

**Background:**

Ischemic vascular diseases are the major cause of death worldwide. In recent years, endothelial cell (EC)-based approaches to vascular regeneration are increasingly viable strategies for treating ischemic diseases, but their applications are challenged by the difficulties in their efficient generation and stable maintenance. Here, we show an alternative protocol that facilitates the generation of functional and expandable ETS variant 2 (ETV2)-induced endothelial-like cells (EiECs) from human adipose-derived stem cells (hADSCs), providing a potential source of cells for autologous ECs to treat ischemic vascular diseases.

**Methods:**

hADSCs were obtained from fresh human adipose tissue. Passage 3 hADSCs were transduced with doxycycline (DOX)-inducible ETV2 transcription factor; purified ETV2-hADSCs were induced into endothelial-like cells using a two-stage induction culture system composed of small molecule compounds and cell factors. EiECs were evaluated for their surface markers, proliferation, gene expression, secretory capacity, and effects on vascular regeneration in vivo.

**Results:**

We found that short-term ETV2 expression combined with TGF-β inhibition is sufficient for the generation of kinase insert domain receptor (KDR)+ cells from hADSCs within 10 days. KDR+ cells showed immature endothelial characteristics, and they can gradually mature in a chemically defined induction medium at the second stage of induction. Futher studies showed that KDR+ cells deriving EC-like cells could stably self-renew and expand about 10^6^-fold in 1 month, and they exhibited expected genome-wide molecular features of mature ECs. Functionally, these EC-like cells significantly promoted revascularization in a hind limb ischemic model.

**Conclusions:**

We isolated highly purified hADSCs and effectively converted them into functional and expandable endothelial-like cells. Thus, the study may provide an alternative strategy to obtain functional EC-like cells with potential for biomedical and pharmaceutical applications.

**Electronic supplementary material:**

The online version of this article (10.1186/s13287-018-1088-6) contains supplementary material, which is available to authorized users.

## Background

Ischemic vascular diseases, such as ischemic heart diseases, ischemic stroke, and end-stage diabetic lower limb ischemia, which are commonly caused by inadequate blood supply and/or impaired functionality of blood vessels, remain the leading cause of death worldwide [1–3]. In the last decade, cell-based therapies have been explored as a treatment option for patients with vascular diseases without option for surgical or endovascular revascularization [4, 5]. Of note, endothelial cell (EC)-based therapies have been shown to be very effective for treating ischemic diseases, with evidence of augmented neovascularization in ischemic tissues not only in animal models but also in many clinical trials [6–8], but limited cell sources and low proliferation restricted their large-scale clinical application and represented one of the major hurdles to overcome for cell-based therapies [9, 10].

Previous studies have reported to generate ECs from pluripotent stem cells (PSCs), including embryonic stem cells (ESCs) and induced pluripotent stem cells (iPSCs), but efficiency of endothelial differentiation, functional integration of converted cells, and the tumorigenic potential of the residual undifferentiated cells are among the challenges to be solved [11, 12]. Recent advances in cell reprogramming technology, i.e., reprogramming of one somatic cell type into another cell type without passing through the pluripotent state, offer new approaches to generate functional ECs [13–15]. It has been reported that direct reprogramming of fibroblasts into ECs can be achieved by forced expression of iPSC-inducing factors (OCT4, SOX2, KLF4, and c-MYC) under defined EC culture conditions [16]. A combination of transcription factors, including ETV2, FLI1, and ERG1 with known roles in regulating broad aspects of endothelial gene expression, was also found effective in inducing functional ECs from amniotic cells [17]. However, human dermal fibroblasts (HDFs) and amniotic cells are not readily accessible cell sources. Several lines of evidences indicate that human adipose-derived stem cells (hADSCs) would be a good choice; adipose tissue is easily obtained in large quantities with little donor site morbidity or discomfort. More importantly, these cells display multipotentiality and can be HLA typed, cryopreserved, and stored for clinical use [18, 19]. Previous studies have described the potential for differentiation of hADSCs into EC-like cells, but these induction methods are time-consuming and ineffective [20, 21].

In this study, by screening developmental signaling factors, we demonstrate that short-term expression of ETV2 in combination with TGF-β inhibition provides for an efficient way to induce hADSCs into proliferative, functional EC-like cells that retain their vascular identity upon serial passaging. This study would be used as a prospective basic work to promote the development of cell therapy and vascular engineering.

## Materials and methods

### Cell culture

Abdominal adipose tissue was obtained from five health donors and umbilical cord was obtained from three health donors in West China Hospital, Sichuan University, upon consent of its donor according to procedures approved by the Medical Ethics Committee, Sichuan University. hADSCs and human umbilical cord mesenchymal stem cells (hUMSCs) were isolated as described [22, 23]. hADSCs and hUMSCs were cultured in mesenchymal stem cell basal medium (DAKEWE, Beijing, China) supplemented with 5% UltraGROTM (HPCFDCRL50, Helios). The complete medium was referred to as MSCM. hADSCs and hUMSCs were characterized as described previously [22, 23]. All of the cells from different donors received EC reprogramming in at least three independent experiments. Human umbilical vein endothelial cells (hUVECs) were isolated from human umbilical veins as described [24] and were cultured in Endothelial Growth Medium-2 (EGM-2; Lonza, USA).

### Lentivirus transduction

The ETV2 lentivirus vectors were purchased from GeneCopoeia company (Guangzhou, China). For lentiviral infection, hADSCs and hUMSCs were dissociated into single-cell suspensions and then replated with lentiviruses and 5 μg/ml polybrene (Sigma-Aldrich, St Louis, MO, USA). After infection for 12 h, the medium was changed with fresh culture medium. Doxycycline (DOX, Sigma D9891) was added to the culture medium, and 1 μg/ml was determined as the optimum concentration of DOX to maintain constant expression of ETV2 in infected hADSCs or hUMSCs.

### Western blotting analysis

Western blotting was performed according to standard procedures elsewhere. The expression of ETV2 in ETV2-hADSCs was detected with anti-human ETV2 mAb (Abcam, Cambridge, MA). GAPDH mAb (Santa Cruz, Biotechnology) was used as an internal control. Following incubation with horseradish peroxidase-conjugated secondary antibody (Zsbio, Beijing, China) at room temperature for 2 h, the bands were detected by a chemiluminescent substrate ECL kit (Merck Millipore).

### Conversion of ETV2-induced ECs (EiECs) from hADSCs

The vascular induction system was described in Fig. 3c. ETV2-transduced hADSCs (ETV2-hADSCs) were plated onto 6-well plates coated with collagen I (BD biosciences) at a density of 5 × 10^4^ cells per well. At the first stage of induction, MSCM was gradually switched into endothelial induction medium (EIM) (with a decreased gradient of MSCM/EIM from 1:1, 1:2, to 1:4), then completely in EIM on day 6 and cultured for another 4 days. EIM contains M199 (Hyclone), 15 mg/ml stem cell-grade BSA (Invitrogen), 1% L-glutamine (Invitrogen), 1% penicillin/streptomycin (Invitrogen), 17.5 μg/ml human insulin (Sigma), 50 μg/ml ascorbic acid (AA) (Sigma), 5 μ/ml heparin (Sigma), and angiogenic growth factors, including 50 ng/ml VEGF (PeproTech), 20 ng/ml bFGF (PeproTech), and 20 ng/ml EGF (PeproTech), which are important for EC development and survival [25]. Additionally, to optimize the induction protocol, modulators of many signal pathways that affect vascular patterning and survival were also evaluated in this system. SB431542 (SB, 10 μM), Chir99021 (3 μM), and BMP4 recombinant protein (20 ng/ml) were included. Five dishes each group of ETV2-hADSCs were prepared; modulator treatment started on days 0, 2, 4, and 6, respectively. The cells cultured in basal medium were used as control. On day 10, cells of each group were collected and KDR expression was analyzed by flow cytometry.

KDR+ cells were sorted 10 days after induction and cultured in endothelial maintenance medium (EMM) with SB (10 μM). In EMM, high concentration of angiogenic growth factors was unnecessary for cell maturation; thus, 20 ng/mL VEGF (PeproTech), 10 ng/mL bFGF (PeproTech), and 10 ng/mL EGF (PeproTech) were applied, and other components remain unchanged. All cell lines were maintained in an incubator (37 °C, 5% CO_2_) with media changes every second day.

### Quantitative real-time PCR (quantitative RT-PCR)

Total RNA was extracted from cells using Trizol (Invitrogen, USA). Quantitative RT-PCR was performed using Step-One Real-Time PCR system (Takara) according to the manufacturer’s instructions. The expression of genes was normalized to β-actin. All reactions were repeated in triplicate, and the primer sequences are listed in Additional file 1: Table S1.

### Immunofluorescence staining and ac-LDL uptake assay

For immunofluorescence staining, the cells were fixed in 4% paraformaldehyde for 20 min at room temperature. Fresh cryosections were fixed on ice-cold acetone for 10 min. Then, the cells or the cryosections were blocked with goat serum and subsequently incubated with primary antibodies at 4 °C overnight. After thorough washing, secondary antibodies were used. Nuclei were visualized with DAPI (Roche Basel, Switzerland). The primary and secondary antibodies are listed in Additional file 1: Table S2. For acetylated low-density lipoprotein (ac-LDL) uptake assay, ac-LDL staining kit was added to the culture media and incubated for 4 h at 37°C according to the manufacturer’s protocol. The results of immunofluorescence staining were determined by HPIAS-100 high-definition color image and text analysis system. Percentage of positive cells was expressed as number of positive cells/number of DAPI-labeled cell nucleus × 100%.

### Microarray analysis

Total RNAs extracted from hADSCs, mature EiECs, and hUVECs were sequenced by the Solexa high-throughput sequencing service (Oebiotech, Shanghai, China). Data were extracted and normalized according to the manufacturer’s standard protocol [26]. The RNAseq raw expression files and details have been deposited in NCBI GEO under accession nos. SRR7072218, SRR7072220, and SRR7072221. Differentially expressed genes were identified using the DESeq (2012) functions estimateSizeFactors and nbinomTest [26]. Genes displaying twofold or greater changes (*P* < 0.05, *t* test) in expression level between hADSCs and mature EiECs were selected to generate the heatmap and for GO term enrichment analysis.

### Human angiocrine factors ELISA

To determine the secretion of human angiocrine factors, mature EiECs, hADSCs, or hUVECs were seeded on 6-well plates and maintained in EIM basal medium without angiogenic growth factors for 48 h until collection of supernatants. Levels of angiocrine factors were measured by the human VEGF ELISA kit (NeoBioscience, EHC108), the human bFGF ELISA kit (NeoBioscience, EHC130), EGF ELISA kit (NeoBioscience, EHC126), IL-8 (NeoBioscience, EHC008), and IGF ELISA kit (R&D, DG100) according to the manufacturer’s instructions. Serum was diluted in a range from 10- to 1000-fold to obtain values falling to the linear range of standard curve.

### Flow cytometry

For the detection of surface markers, cells were dissociated into single-cell suspension and resuspended in PBS and then stained with fluorochrome-labeled mAbs for 30 min on ice in the dark. The flow cytometry analysis was performed using a flow cytometer (Beckman Coulter, Fullerton, CA, USA) or a BD Bioscience Influx cell sorter; collected events were analyzed by FlowJo software (Treestar, Ashland, OR, USA). The antibodies (all from Biolegend) are listed in Additional file 1: Table S2.

### Capillary-like structure formation assay

To assess the formation of capillary structures, tested cells were trypsinized into single cells and resuspended in EGM-2 medium supplemented with 50 ng/ml VEGF. Cells were plated at a density of 5 × 10^4^ cells per well in triplicate in 24-well plates coated with growth factor-reduced Matrigel (BD Biosciences), plates were incubated overnight, and tube formation was observed by phase-contrast microscope. The amount of branch points (≥ 3 cells per branch) were counted and analyzed in five random fields per replicate.

### In vivo Matrigel angiogenesis assay

To assess the angiogenesis potency of EiECs in vivo, about 1 × 10^6^ EiECs were suspended in 100 μl PBS containing 30% Matrigel and injected subcutaneously into the athymic nude mice (*n* = 5). Two weeks after implantation, the cell masses were taken out and observed. hADSCs and hUVECs were used as controls.

### Hind limb ischemic mouse model and angiogenesis assay

All the animal care and experiments were approved by the Animal Care and Use Committee of Sichuan University. Hind limb ischemic experiments were performed as previously described [27]. Briefly, 8-week-old male athymic nude mice (Beijing Vitalstar Biotechnology Co., Ltd.) were anesthetized with 10% chloral hydrate (Sigma). The unilateral femoral artery and its branches were ligated through a skin incision with 6–0 silk (Ethicon). The femoral artery was excised from its proximal origin to the distal point where it bifurcates into the saphenous and popliteal arteries. Immediately after the surgery, mice were injected with 1 × 10^6^ cells (suspended in 100 μl PBS containing 30% Matrigel) at three equally spaced points on the adductor muscle of the ischemic thigh (*n* = 10); PBS containing 30% Matrigel served as negative control. The physiological status of the ischemic limbs was assessed according to the ischemia score index previously reported [28]:0, no changes; 1, discoloration/necrosis of the nails; 2, necrosis of the toes; 3, foot necrosis; 4, leg necrosis (up to the gastrocnemius muscle); and 5, autoamputation; to sum up, the physiological status of mouse limb was assessed as limb salvage (scored 0 to 2) or limb necrosis (scored 3 to 4) and the worst, 5 points, represented limb loss. In addition, hind limb blood flow was determined weekly using the laser Doppler imaging (Laser Doppler Perfusion Imager System, Moor Instruments); limb perfusion was expressed as the ratio of ischemic to non-ischemic hind-limbs. Then, five mice in each group were euthanized; the ischemic limbs of the mice were dissected for cryosectioning. Blood vessels labeled with mouse/human CD31 or human-specific CD31 in sections were counted respectively in five randomly chosen fields. Blood vessels were identified as lumenal structures. Microvessel density was reported as the average number of microvessels and expressed as blood vessels per square millimeter. Percentage of human-specific CD31+ vessels was expressed as human-specific CD31+ vessels/total vessels × 100%. Cell tumorigenicity was evaluated on the rest of the mice over 4 months.

### Statistics

Values are obtained from at least three independent experiments and reported as the mean ± SEM. Comparisons between the groups were performed by a one-way analysis of variance (ANOVA) with Tukey’s multiple comparison or two-way analysis of variance (ANOVA) with Tukey’s multiple post comparison. Statistical significance was accepted at *P* < 0.05. All graphs were plotted with GraphPad Prism software.

## Results

### Efficient endothelial induction from hADSCs by ETV2 transduction

Untransduced hADSCs maintained spindle-shaped, fibroblast-like appearance (Additional file 2: Figure S1A). They were positive for the known markers CD29, CD44, CD73, CD90, CD105, and CD166 and negative for the specific markers of ECs and hematopoietic stem cells including CD31, CD34, and CD45 (Additional file 2: Figure S1B). Furthermore, hADSCs showed the multipotency when cultured in osteo-, adipo-, and chondrogenic differentiation medium (Additional file 2: Figure S1C-E). After identification, hADSCs (passage 5) were selected for experiments. To determine whether hADSCs could be converted into ECs by ETV2 transduction, we cloned a doxycycline (DOX)-inducible lentiviral construct containing the open reading frame of ETV2 gene. After transducing into hADSCs, expression of ETV2 in response to DOX treatment was confirmed by immunofluorescence staining, quantitative RT-PCR, and Western blotting (Additional file 2: Figure S2A-C).

Transduced ETV2-hADSCs subsequently cultured in chemically defined endothelial induction medium (EIM). As early as 5 days later, multiple endothelial developmental factors, including ERG, FOXC1, FOXC2, GATA2, TAL1, and FLI1, were upregulated in ETV2-hADSCs and peaked around day 7 to day 10, which is indicative of the fact that cells enter the endothelial fate (Additional file 2: Figure S3A). By contrast, the pluripotency-associated genes for Oct4, Sox2, and Nanog and the early mesodermal genes for Brachyury, GSC, and MIXL-1 remained undetected (Additional file 2: Figure S3B-C), suggesting that ETV2 expression in hADSCs directly induced expression of endothelial genes without passing through the pluripotent and early mesodermal stages.

**Fig. 1:**
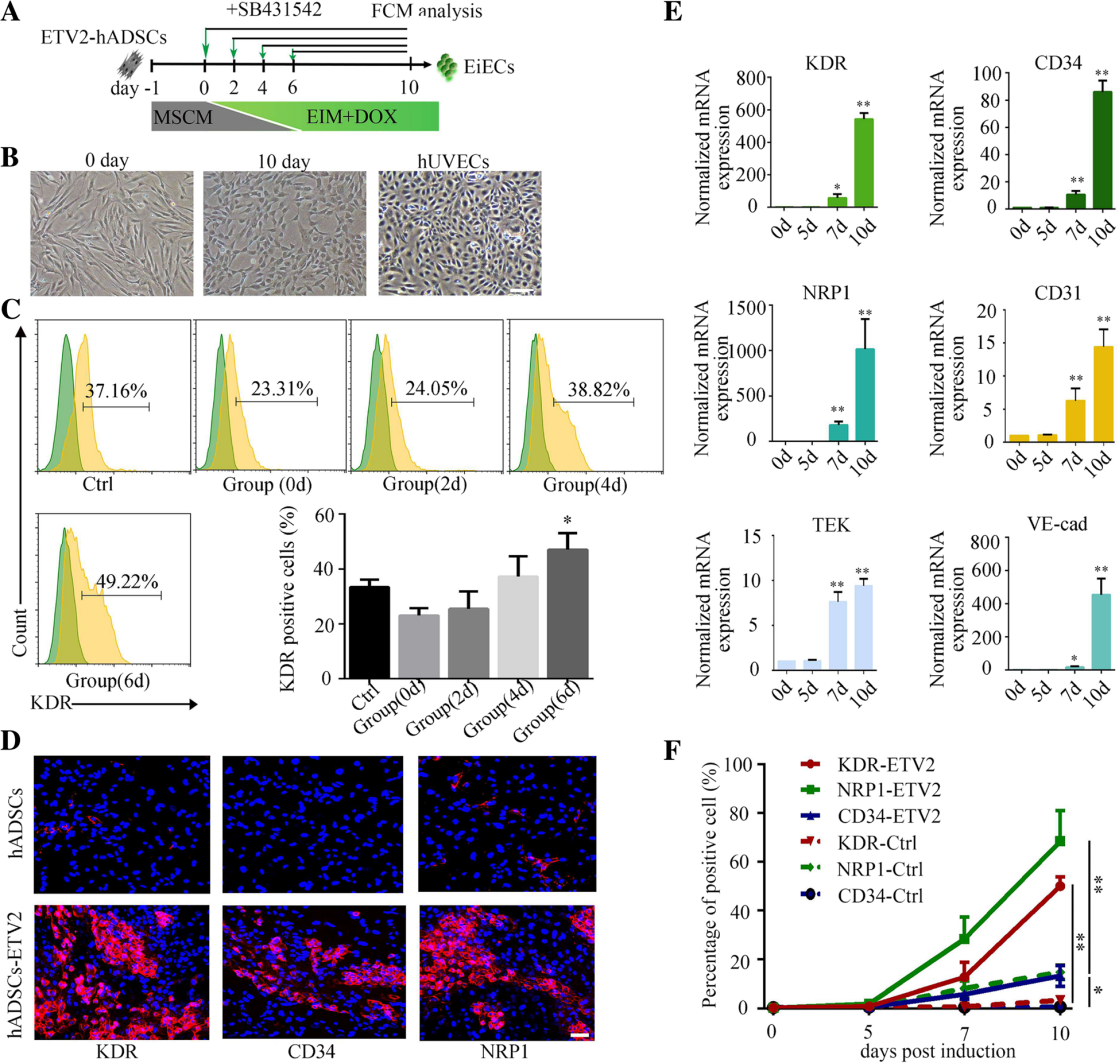
Efficient endothelial induction from hADSCs by ETV2 transduction. **a** Schematic of the induction protocol. **b** Induced ETV2-hADSCs exhibited cobblestone appearance during EC induction. **c** Representative flow cytometric analysis of KDR+ cells in ETV2-hADSCs that treated with SB431542 (SB) at indicated time points. Cells cultured in EIM were treated as control. **d** Expression of endothelial-specific markers KDR, CD34, and NRP1 were measured by immunofluorescence staining 10 days post induction. Scale bar = 50 μm. **e** mRNA levels of endothelial-specific genes were determined by quantitative RT-PCR in ETV2-hADSCs at the indicated time points. Data are normalized to β-actin. **f** Endothelial-specific markers KDR, NRP1, and CD34 in ETV2-hADSCs were measured by flow cytometry during induction. *P < 0.05, **P < 0.01

To improve induction efficiency and stabilize the endothelial fate, modulators of many pathways affecting vascular patterning and survival [29], including the Wnt, BMP4, and TGF-β pathways, were assessed in this induced system (Fig. 1a and Additional file 2: Figure S4A). As a critical marker of early vascular progenitors [30, 31], the expression of KDR was used to evaluate the generation of EC-like cells from hADSCs. After a series of experiments, we found that inhibition of TGF-β signaling with SB431542 (SB) from day 6 of induction combined with continuous DOX treatment resulted in a cobblestone appearance (Fig. 1b and Additional file 2: Figure S3D) and dramatically enhanced KDR expression in the majority of the generated cells (49.2 ± 1.9%), which means nearly half of induced cells with endothelial features 10 days after induction (Fig. 1c). However, replenishment of Chir99021 and BMP4 recombinant protein which activates Wnt and BMP4 pathway respectively or exposure to SB before day 6 of induction could not increase the proportion of KDR+ cells (Additional file 2: Figure S4B-C and Fig. 1c). After culturing in the final optimized conditions, expression of other early endothelial lineage-specific genes, like NRP1 and CD34, was highly upregulated during induction (Fig. 1d–f). However, the genes expressed in mature endothelial cells, including CD31 and TEK, were not upregulated at this stage of induction. These results demonstrated stage-specific TGF-β inhibition synergized with ETV2 could efficiently promote the generation of early EC-like cells from hADSCs. In summary, our studies identify an induction system leading to the generation of early ECs in an efficient manner.

### KDR-positive cells serve as early EC-like cells and have the capability for vessel formation

**Fig. 2:**
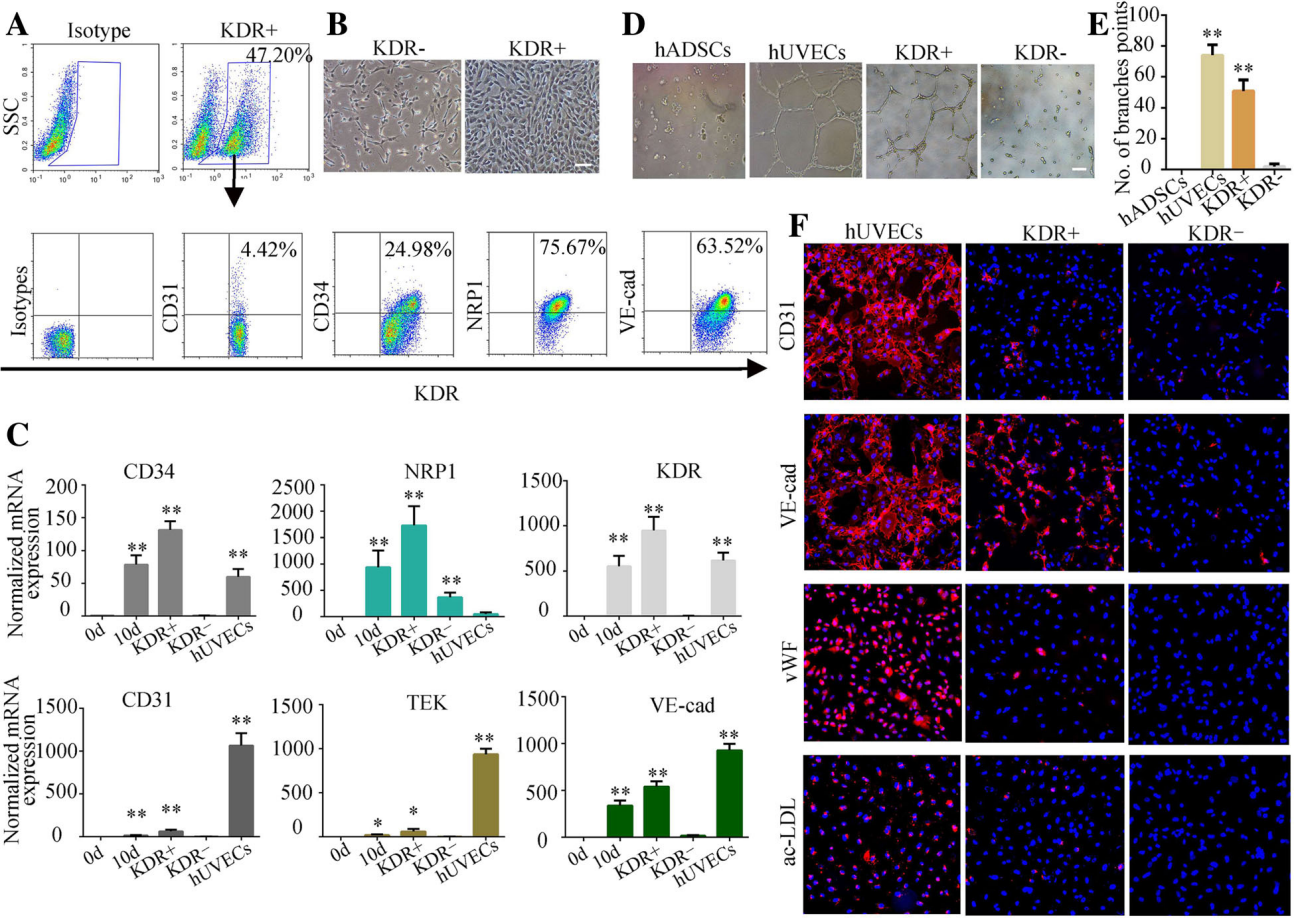
Isolation and characterization of KDR+ cells. **a** Phenotypic analysis of KDR+ cells after sorting. **b** Typical cell morphology of KDR− cells (left) and KDR+ cells (right). **c** mRNA levels of endothelial-specific genes were determined by quantitative RT-PCR on induced ETV2-hADSCs at day 10; the results of unsorted cells and the purified KDR+ and KDR− cells were shown. Data are normalized to β-actin. **d** KDR+ cells, similar to hUVECs, were able to form tubular structures on Matrigel-coated plates. **e** The number of branches were quantified (*n* = 5). **f** Immunofluorescence of endothelial markers CD31, VE-cad, vWF, and ac-LDL uptake were detected in KDR+ and KDR− cells; hUVECs were used as positive control. Scale bar = 50 μm. *P < 0.05, **P < 0.01

KDR was reported as a marker to isolate vascular progenitor cells from human embryonic stem cells (hESCs) grown in endothelial differentiation medium [30, 31]. To identify whether ETV2-induced KDR+ cells represent a developmental state similar to that of early ECs, we sorted the heterogeneous cells by flow cytometry based on the expression of KDR. As shown in Fig. 2a, b, two distinct populations, KDR+ and KDR−, with different cell morphologies were detected at 10 days post-induction. Compared to KDR− cells, KDR+ cells coexpressed high levels of NRP1 (75.7 ± 7.1%) and VE-cad (63.5 ± 6.2%) and moderate levels of CD34 (25 ± 3.2%) and CD31 (4.4% ± 3.6%) (Fig. 2a), while endothelial-specific genes, including NRP1, VE-cad, CD34, and CD31 were expressed at low level in KDR− cells (Additional file 2: Figure S5). Additionally, the expression level of these markers in KDR+ and KDR− cells was also confirmed at the mRNA level (Fig. 2c).

In order to evaluate the capability for vessel formation, purified KDR+ or KDR− cells were trypsinized into single cells and resuspended in EGM-2 medium with 50 ng/ml VEGF, hADSCs, and hUVECs used as controls. Notably, compared with KDR− cells, KDR+ cells aggregated into vessel-like structures and formed more branch points on Matrigel-coated plates, in a manner similar to hUVECs (Fig. 2d, e). Compared to hUVECs, immunofluorescence staining showed that VE-cad was highly expressed, while CD31 and vWF were lowly expressed in KDR+ cells (Fig. 2f), and only a few KDR+ cells were capable of acylated low-density lipoprotein (ac-LDL) uptake (Fig. 2f). These data indicated that the KDR+ cells represent putative early EC-like cells derived from ETV2-hADSCs.

### Stage-specific ETV2 downregulation augments generation of mature and stable EiECs

**Fig. 3:**
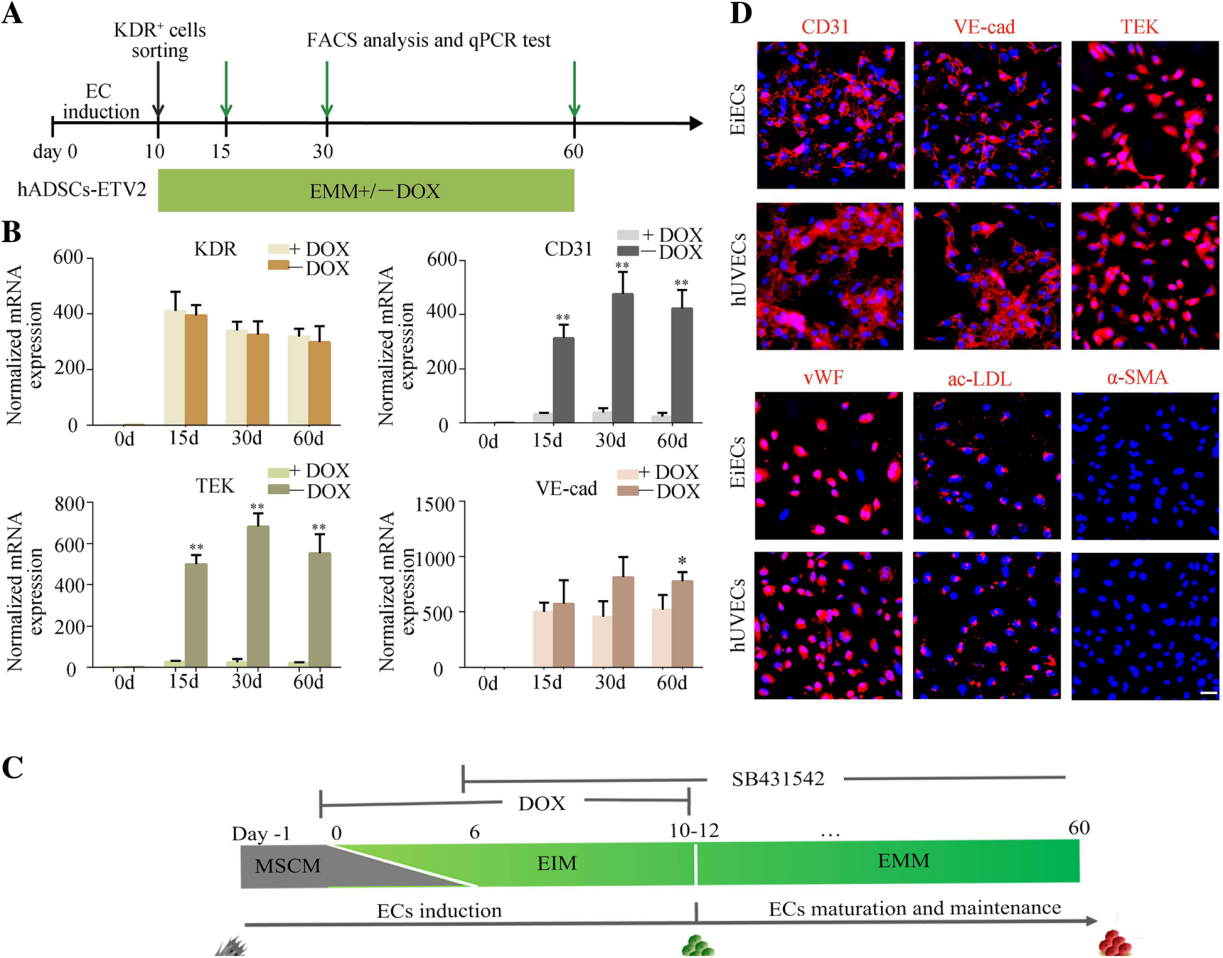
Stage-specific ETV2 downregulation augments generation of mature and stable EiECs from KDR+ cells. **a** Schematic representation of the two-stage conversion process. **b** Sorted KDR+ cells cultured in endothelial maintenance medium (EMM) with or without DOX were detected by quantitative RT-PCR at different time points. Quantitative RT-PCR data are normalized to β-actin. **c** The final optimal scheme of direct endothelial reprogramming from transduced ETV2-hADSCs. *P < 0.05, **P < 0.01. **d** Immunofluorescence of endothelial cell markers CD31, VE-cad, vWF, and ac-LDL uptake were detected in EiECs; hUVECs were used as positive control. Scale bar = 50 μm

In the present study, despite the generation of KDR+ early endothelial populations from hADSCs, only a few mature ECs were developed after culturing for additional time with DOX (Fig. 2c, e). Previous work has shown ETV2 is minimally or not expressed in mammalian postnatal ECs [32, 33]. To promote maturation of KDR+ cells, we removed DOX from the endothelial maintenance medium (EMM) in the second stage (after day 10) of induction. Cells with persistent DOX treatment were served as control (Fig. 3a). Notably, compared with control cells, downregulation of ETV2 expression effectively increased the expression of endothelial markers, including CD31, VE-cad, and TEK (Fig. 3b). Moreover, analysis by quantitative RT-PCR revealed switching off ETV2 in KDR+ cells did not interfere with expression of downstream EC-specific ETS family transcription factors, FLI-1 and ERG, which are essential for specification of functional ECs (Additional file 2: Figure S6). These results indicated downregulation of ETV2 was beneficial for the generation of ETV2-induced ECs (EiECs). The final optimized protocol is described in the “Materials and methods” section and summarized in Fig. 3c.

**Fig. 4:**
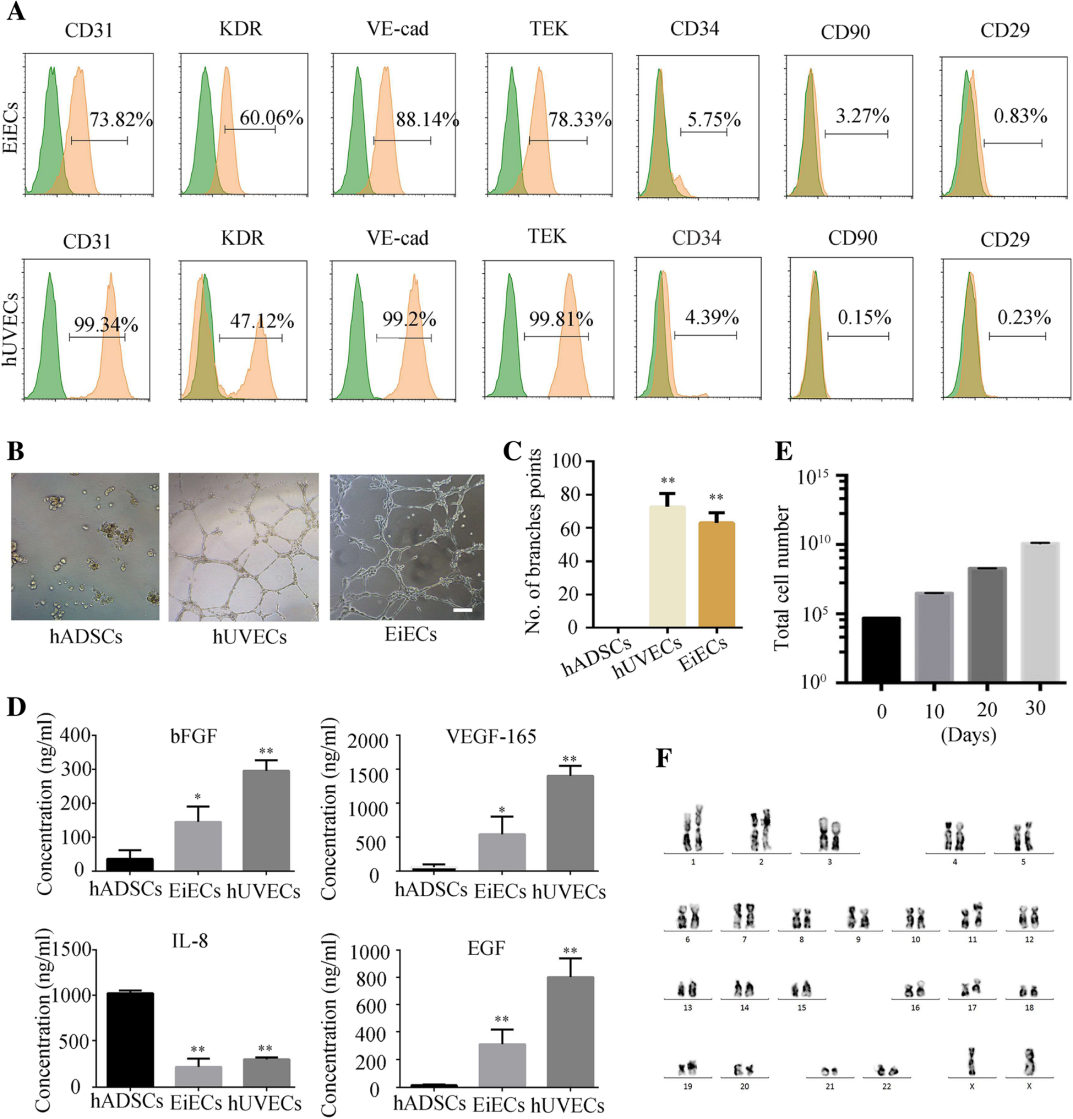
Characterizations of EiECs in vitro*.* **a** Representative flow cytometric analysis of EiECs and hUVECs. **b** EiECs, similar to hUVECs, were able to form tubular structures on Matrigel-coated plates. **c** The number of branches were quantified (*n* = 5). Scale bar = 50 μm. **d** Quantitative analysis of angiocrine factor secretion among hADSCs, EiECs, and hUVECs by ELISA. **e** Growth curves of EiECs during long-term expansion in EMM with SB. **f** Cells were collected at nine passages, G banding analysis of EiECs demonstrating a normal human karyotype (44, XX). *P < 0.05, **P < 0.01

To further evaluate the functional abilities of EiECs, we collected these cells on day 30 of induction and compared them to hUVECs. Immunofluorescence staining and flow cytometry analysis revealed the EiECs were positive for endothelial-specific markers, including VE-cad, CD31, TEK, and vWF (Fig. 3d and Fig. 4a). Functionally, EiECs were capable of ac-LDL uptake (Fig. 3d) and could form capillary-like structures on Matrigel-coated plates (Fig. 4b, c). In addition, EiECs secreted many angiocrine factors, including VEGF, bFGF, and EGF (Fig. 4d). Moreover, EiECs could proliferate extensively for more than nine passages in 1 month, with 5 × 10^4^ starting cells robustly producing about 1.2 × 10^10^ EiECs (Fig. 4e) with a normal karyotype (Fig. 4f). These results, along with the aforementioned findings, confirmed that EiECs derived from KDR+ cells represent functional and proliferative mature EC-like cells.

### Mature EiECs display a transcriptome profile that is similar to mature ECs

**Fig. 5:**
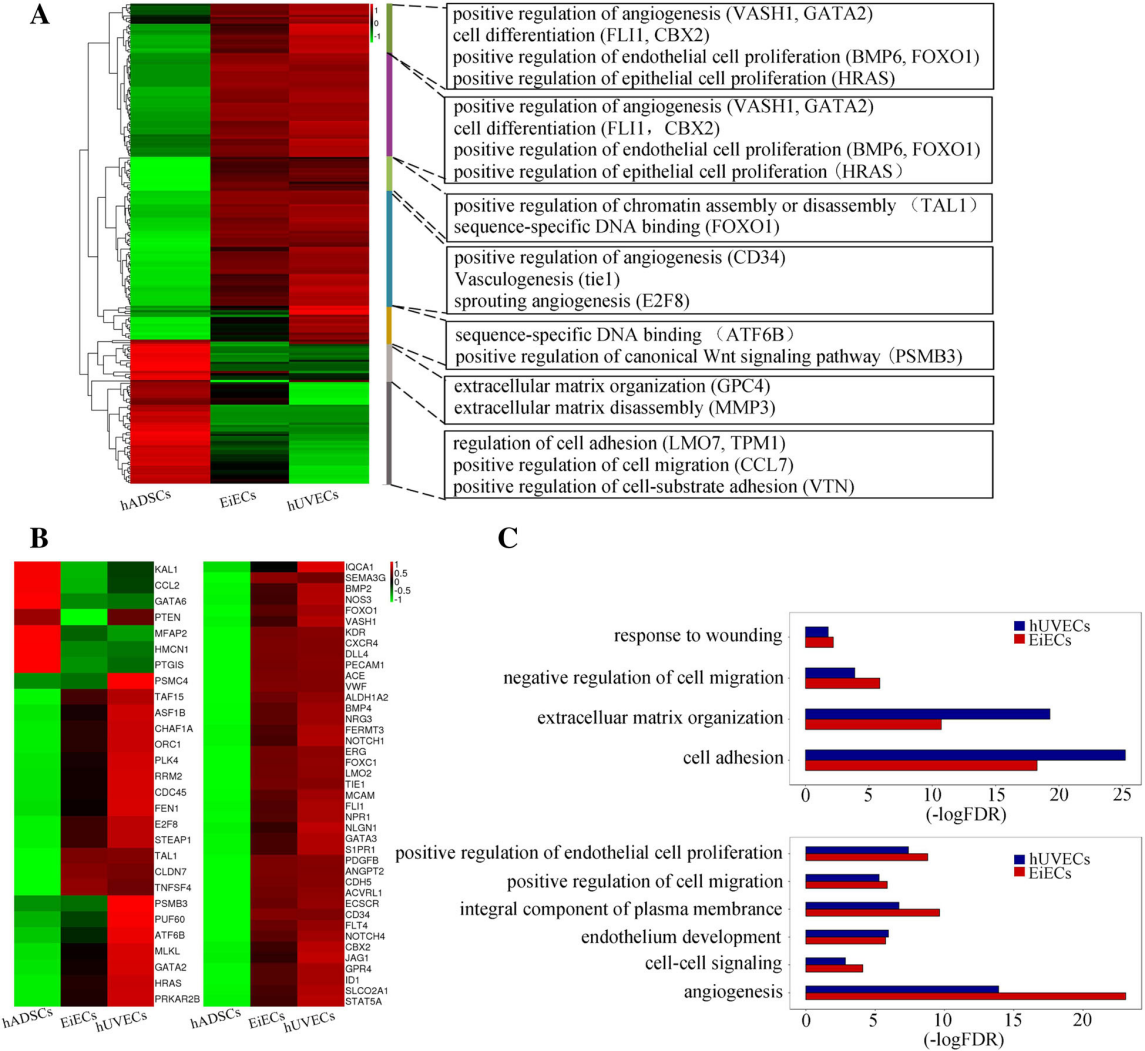
Transcriptome analysis on mature EiECs via RNA sequencing. **a** Transcriptome analysis revealing differences in gene expression among hADSCs, mature EiECs, and hUVECs detected by RNA-seq. Representative gene ontology (GO) terms and gene symbols are also shown in the right. **b** Heatmap representing EC-related genes with twofold difference. Expression values relative to the average expression values across all samples were represented by colors from green to red (log2 scale). **c** GO analyses of downregulated (up) and upregulated (down) genes in mature EiECs/hUVECs

The global gene expression patterns of hADSCs, hUVECs, and mature EiECs were further analyzed by microarray. Compared to hADSCs, 577 genes were upregulated and 505 downregulated by more than twofold in mature EiECs. Microarray data revealed numerous vascular genes were upregulated in mature EiECs compared to naive hADSCs (Fig. 5a). Furthermore, the expression levels of these induced EC genes approached those seen in hUVECs, supporting that mature EiECs have attained a relatively complete EC identity (Fig. 5a). To gain more detailed insight into the transcriptome profiles of hADSCs, hUVECs, and mature EiECs, 69 EC-related genes were analyzed. We found mature EiECs displayed a significant induction in EC-related genes, including KDR, CD34, and CD31 in a manner more similar to hUVECs than hADSCs (Fig. 5b). Gene ontology term enrichment analysis of these genes indicated genes involved in angiogenesis, positive regulation of EC proliferation, cell-cell signaling, and endothelium development were similar to hUVECs (Fig. 5c), indicating a clear transition from an hADSC-like to EC-like state. Therefore, genome-wide analyses demonstrated mature EiECs resemble mature ECs in terms of global gene expression.

#### Transplantation of EiECs into nude mice resulted in formation of microvessels

**Fig. 6:**
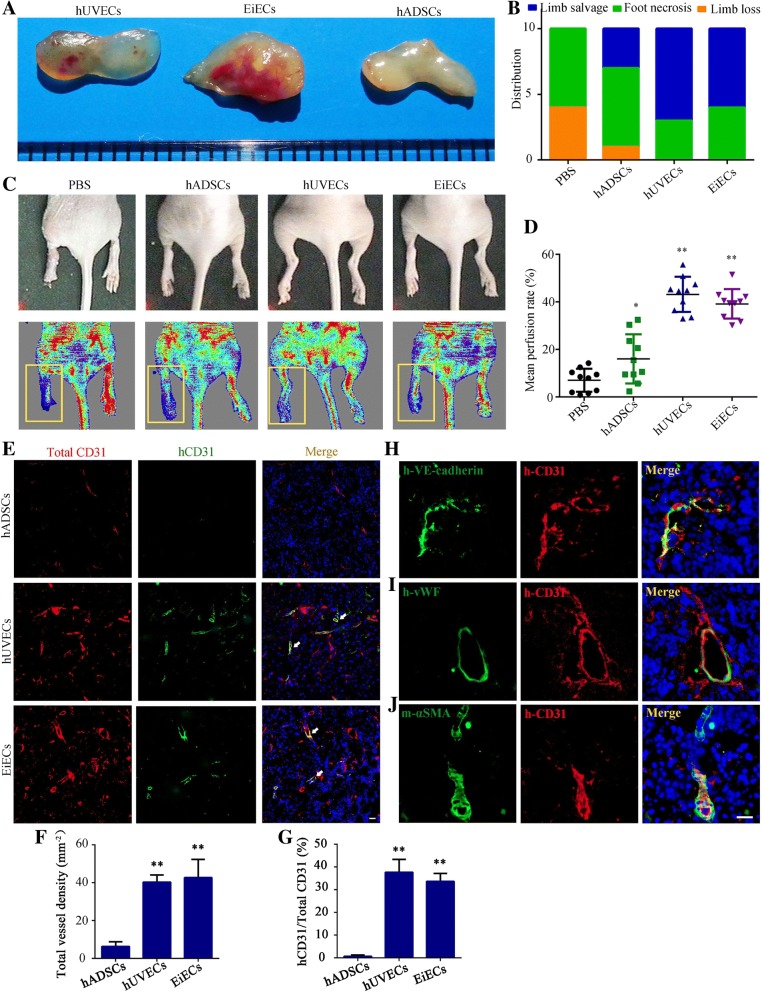
EiECs promote revascularization in ischemic limbs of athymic nude mice. hUVECs, mature EiECs, or hADSCs were implanted subcutaneously into nude mice. **a** Cell masses were removed and imaged 14 days after implantation. **b** hUVECs, mature EiECs, or hADSCs were injected into adductors of an ischemic model of nude mice; PBS containing 30% Matrigel was used as control. Stacked bar graph shows the percentage distribution of the physiological status of the ischemic limbs 4 weeks after cell transplantation. **c** Representative Doppler images of superficial blood flow in lower limbs. The yellow box indicated the left ischemic hind limbs. **d** Quantitative analysis of perfusion recovery in ischemic hind limbs. **e** Sections of Matrigel plugs were stained with mouse/human CD31 antibody (red) to identify the functional vessels. Human-specific CD31 was stained in green; white arrows indicate the colocalization of human CD31 and mouse CD31-marked vessels. **f**, **g** Vessel density and percentage of human-specific CD31+ vessels were also quantified. **h**–**j** Panels showed individual and merged images of immunofluorescence analysis performed on Matrigel plugs. **h** Colocalization of human CD31 (red) and human-VEcad (green) or (I) human-CD31 (red) and human-vWF (green) were observed. **j** The bottom row shows fields containing human-CD31-positive cells (red) as part of the endothelial layer surrounding staining for mouse specific a-SMA (green). Scale bar = 50 μm. *P < 0.05, **P < 0.01

To investigate the effects of EiECs on vascular regeneration in vivo, we subcutaneously implanted 1 × 10^6^ EiECs, hUVECs, or hADSCs into the dorsal region of nude mice (*n* = 5). Fourteen days post-implantation, the cell masses were removed and imaged. Compared to the hADSC group, more macroscopic vessels had assembled in EiEC and hUVEC groups (Fig. 6a). To assess the therapeutic potential of EiECs in ischemic tissue, we conducted intramuscular injection of solution-suspended EiECs, hUVECs, or hADSCs into the ischemic adductor muscles of nude mice that had been subjected to femoral artery ligation (*n* = 10). Control mice were injected with an equivalent volume of solution, and hind limb blood flow recovery was monitored over 30 days. The control mice displayed rapid and extensive muscle necrosis in their ischemic hind limbs, resulting in severe limb necrosis (60%, 6/10) or complete limb loss (40%, 4/10). Similarly, nearly 60% of the mice receiving a hADSC transplant suffered from limb necrosis and one mouse ultimately lost its limb. By contrast, transplantation of EiECs protected the ischemic muscles from necrosis, where the limbs could be salvaged in more than 60% of mice (Fig. 6b). Consistent with the physiological status of the ischemic limbs, quantitative analysis by laser Doppler monitoring revealed a significant improvement in the mean perfusion ratio after transplantation of mature EiECs, which peaked 14 days post-delivery (0.37 ± 0.06), compared to control mice (0.11 ± 0.07) and mice injected with hADSCs (0.18 ± 0.07) (Fig. 6c, d).

To further verify engraftment of transplanted EiECs in the ischemic tissues, we also performed histological analysis 14 days post-transplantation. Using a CD31 antibody that was cross-reactive to human and mice (total CD31), we first stained total vessels in the ischemic tissue slices (Fig. 6e). Further analysis with human-specific CD31 antibody demonstrated mice transplanted with EiECs and hUVECs, but not hADSCs, developed human CD31-expressing blood vessels in their ischemic limbs (Fig. 6e). The histological findings were confirmed by a quantitative analysis, which showed that blood vessel density in the hUVEC-implanted and EiEC-implanted group was nearly eightfold higher than that in the hADSC-implanted group and hUVECs or EiECs constituting vessels accounting for 30–40% of the total vessels (Fig. 6f, g).

In addition, the human CD31 expressing EiEC vessels also expressed human-specific VE-cad (Fig. 6h) and vWF (Fig. 6i), and a portion of the vessels were stabilized by connections with mouse α-smooth muscle actin (α-SMA)-positive pericytes (Fig. 6j). These histological data indicated EiECs survived in ischemic muscles and formed functional blood vessels that anastomosed to the host vasculature. Importantly, none of the EiEC recipients formed tumors after a long observation (4 months) (Additional file 2: Figure S7). Altogether, our results demonstrated EiECs have the capacity to promote revascularization in ischemic regions and are safe in potential therapeutic applications.

### Efficient endothelial induction from human umbilical cord-derived mesenchymal stem cells by ETV2 transduction

**Fig. 7:**
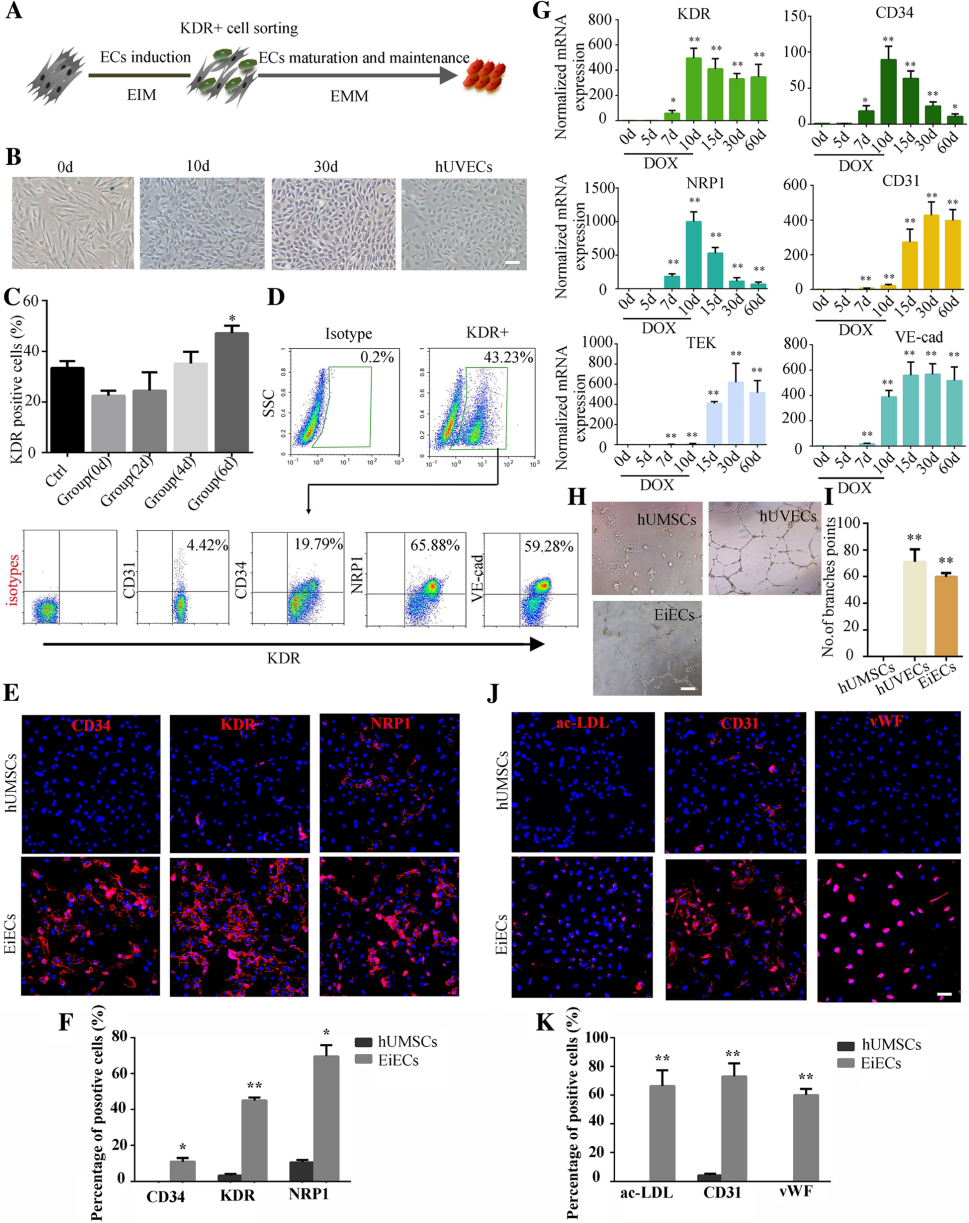
Efficient endothelial induction from hUMSCs by ETV2 transduction. **a** A schematic of the optimal induction protocol. **b** Induced ETV2-hUMSCs exhibited cobblestone appearance during EC induction. **c** Quantitative and representative flow cytometric analysis of KDR in induced ETV2-hUMSCs treated with SB at indicated time points; cells cultured in basal medium were treated as control. **d** Phenotypic analysis of KDR+ cells after fluorescence-activated cell sorting. **e**, **f** Immunofluorescence showed expression of KDR, CD34, and NRP1 in induced ETV2-hUMSCs on 10 days post-induction. **g** mRNA levels of endothelial-specific genes were determined by quantitative RT-PCR in induced ETV2-hUMSCs at the indicated experimental time points. Data are normalized to β-actin. **h** EiECs derived from induced ETV2-hUMSCs were able to form tubular structures on Matrigel-coated plates. **i** The number of branches were quantified (*n* = 5). **h**–**k** Immunofluorescence of endothelial markers CD31, vWF, and ac-LDL uptake were detected in mature EiECs; hUVECs were used as positive control. Scale bar = 50 μm. *P < 0.05, **P < 0.01

To determine whether functional EiECs could be induced from other mesenchymal stem cell (MSC) sources, we isolated three samples of human umbilical cord-derived mesenchymal stem cells (hUMSCs) and infected them with lentivirus harboring the DOX-inducible transgene encoding ETV2. After culturing these cells in the same endothelial induction conditions used for hADSCs (Fig. 7a), cobblestone-like morphologic emerged in induced ETV2-hUMSCs (Fig. 7b). Similar to observations with hADSCs, exposure to SB from day 6 of induction could further improve the efficiency of KDR+ cell production (Fig. 7c). Analysis by flow cytometry determined these KDR+ cells were positive for other endothelial lineage markers, including NRP1 (65.9 ± 6.3%), CD34 (19.8 ± 2.2%), and VE-cad (59.3 ± 3.9%) (Fig. 7d); these results were further confirmed by immunofluorescence staining (Fig. 7e, f). When cultured in the EMM in the absence of DOX from day 10, hUMSC-derived KDR+ cells gradually converted into mature endothelial lineage cells with the efficiency comparable to hADSCs (Fig. 7g). Furthermore, these hUMSC-derived EiECs could form capillary-like structures in vitro (Fig. 7h, i), expressed EC markers CD31 and vWF, and were capable of ac-LDL uptake (Fig. 7h–k). These results suggested MSCs from different origin tissues could also be reproducibly induced into functional EiECs using this defined induction system.

## Discussion

Here, we demonstrate that short-term ETV2 expression and TGF-β inhibition can stably convert hADSCs into functional EC-like cells in a chemically defined induction medium. These cells exhibit many typical endothelial features, including their endothelial morphology, expression of endothelial-specific markers, basic functional properties of ECs, and global gene expression patterns. Early EC-like cells and EiECs would be useful to model in drug discovery and in the analysis of the pathogenesis of human vascular diseases. EiECs would also be a potential cell source for cell therapy and vascular tissue engineering.

Among the ETS factors regulating vascular development, recent attention has focused on ETV2 (also known as ER71) based on its critical role in endothelial specification [32, 34]. In this study, we found that short-term ETV2 expression was sufficient to induce expression of multiple key EC development-associated factors, such as FLI1 and ERG, as well as a high number of genes associated with early endothelial lineages, including CD34, KDR, and NRP1 during EC induction from hADSCs. The platform that we used further supports an important role of ETV2 as the core factor in EC development, and this study provides the first evidence of ETV2-mediated direct converting of MSC, including hADSCs and hUMSCs, into early EC-like cells in the chemically defined induction medium.

Recent studies have reported activation of TGF-β signaling favors endothelial-to-mesenchymal transition, which impedes the generation of endothelial progenitor cells and abrogates KDR expression in ECs [35, 36]. Supported with these reports, we found that inhibition of TGF-β signaling with SB431542 (SB) could further increase the proportion of KDR+ cells to nearly 50% of differentiated ETV2-hADSCs, proving to be a more effective approach than previous studies [37–39], which reported 3.1%, 7.5%, and 20% ECs generated from human pluripotent stem cells or human fibroblasts. However, in our study, KDR expression was inhibited if cells were prematurely exposed to SB, suggesting the biphasic role of TGF-β signaling in vasculogenesis (i.e., it stimulates endothelial commitment from hADSCs in the early phase but inhibits endothelial development in the late stage of induction) [40]. Remarkably, our data also demonstrated SB as a key stimulus for the proliferation of EiECs, probably by reducing endothelial-to-mesenchymal transition, upregulating Id1 expression, or stabilizing KDR expression [17, 41]. Thus, induction of hADSCs not only requires enforced expression of TFs, but also requires modulation of signaling pathways to establish stable EiECs.

Another important finding is the long-term expansion of EiECs in chemically defined medium. For cell-based therapies, it is highly desirable to generate expandable sources of committed cells. Previous studies reported that EC-like cells can be induced from MSCs. However, expansion of these cells in therapeutically large numbers is technically challenging. Our results demonstrated that EiECs were stable over prolonged culture (nine passages) in chemically defined conditions (nearly 1 × 10^6^-fold expansion) with a normal karyotype. Our results may enable highly efficient production of patient-derived ECs for the treatment of vascular diseases.

Cell-based therapy is a promising approach for treating a range of ischemic diseases. hADSCs have already been used in clinical trials of ischemic diseases [19, 42]; however, previously published results have revealed considerable uncertainty about the true clinical potential of hADSCs for ischemic diseases [43]. Consistent with these results, our studies using hADSCs found generation of significantly fewer vessels following subcutaneous implantation, as well as less efficient revascularization compared to hUVECs and EiECs. These results suggest the therapeutic potential of pure hADSCs may be limited for ischemic diseases. Indeed, we found that implantation of hADSC-derived EiECs could provide long-term functional improvement (4 months) in the ischemic limbs of mice. This beneficial effect may result from the contribution of EiECs to therapeutic vascularization that aids in supplying oxygen and nutrients to ischemic tissues. In addition, this improvement may also result from reparative effects from paracrine factors bFGF, VEGF, and EGF secreted by EiECs. Notably, no tumors were found in any recipients during the 4-month experimental time frame. These results highlight the safety and therapeutic utility of hADSC-derived ECs in repairing tissue ischemia and enhancing neovascularization in vivo. Although effective in inducing EiECs, viral vector-carried transcription factor is still not favorable in therapeutic application; the use of nonintegrating genetic delivery, such as episomal vectors, mRNA, or small molecule compound, could avoid potential issues associated with viral integration. Further studies are needed to understand the therapeutic mechanisms that occur after EiEC transplantation, and improvement to enhance the engraftment of EiECs is anticipated.

In conclusion, our results showed that functional and expandable ECs can be derived from hADSCs with only ETV2 factor, when combined with the appropriate inductive signals. This study would be used as a prospective basic work to promote the development of vascular engineering and drug discovery. Furthermore, future mechanistic studies based on regulation of EC-specific TFs will also help us gain insights into the roles of the related signaling pathways in vascular regeneration.

## Additional files

Additional file 1: **Table S1.** Quantitative RT-PCR primer sequences. Table S2 Antibodies used for immunofluorescence and FACS analysis. (DOCX 19 kb)

Additional file 2: **Figure S1.** Characterization of hADSCs. **Figure S2.** Characterization of ETV2-hADSCs. **Figure S3.** ECs were generated from ETV2-hADSCs did not through pluripotent and early mesoderm stage. **Figure S4.** Investigation of signaling molecules of important pathway in regulating EC-fate conversion. **Figure S5.** Isolation and characterization of KDR−- cells. **Figure S6.** Downregulation of ETV2 promotes the maturation of KDR+ cells. **Figure S7.** Long-term blood recovery and tumorigenic assessment of mature EiECs in vivo. (DOCX 18378 kb)

## Abbreviations

ac-LDL: Acetylated low-density lipoprotein
DOX: Doxycycline
ECs: Endothelial cells
EGM-2: Endothelial Growth Medium-2
EiECs: ETV-induced endothelial-like cells
EIM: Endothelial induction medium
EMM: Endothelial maintenance medium
ESCs: Embryonic stem cells
ETV2: ETS variant 2
hADSCs: Human adipose-derived stem cells
HDFs: Human dermal fibroblasts
hUMSCs: Human umbilical cord mesenchymal stem cells
hUVECs: Human umbilical vein endothelial cells
iPSCs: Induced pluripotent stem cells
KDR: Kinase insert domain receptor
PSCs: Pluripotent stem cells
SB: SB431542
