# Supplementary material for: Conversion of human adipose-derived stem cells into functional and expandable endothelial-like cells for cell-based therapies
Source: Stem Cell Res Ther. 2018 Dec 17;9:350. doi: 10.1186/s13287-018-1088-6 (PMC6296081; doi:10.1186/s13287-018-1088-6)
Supplement: Supplementary file 1 — Table S1. Quantitative RT-PCR primer sequences. Table S2 Antibodies used for immunofluorescence and FACS analysis. (DOCX 19 kb) [file 13287_2018_1088_MOESM1_ESM.docx]

**Table S1. Quantitative RT-PCR primer sequences**

| Gene name | Sequence (5’ to 3’) |
| --- | --- |
| OCT-4 | CTTCTGCTTCAGGAGCTTGG  GAAGGAGAAGCTGGAGCAAA |
| SOX2 | GGGAAATGGGAGGGGTGCAAAAGAGG  TTGCGTGAGTGTGGATGGGATTGGTG |
| NANOG | CCTATGCCTGTGATTTGTGGG  AGGTTGTTTGCCTTTGGGAC |
| Brachyury | CAGTGGCAGTCTCAGGTTAAGAAGG  CGCTACTGCAGGTGTGAGCAA |
| GSC | AACGCGGAGAAGTGGAACAAG  CTGTCCGAGTCCAAATCGC |
| MIXL-1 | GTACCCCGACATCCACTTGC  AGGATTTCCCACTCTGACGC |
| FOXC2 | ACAGCTACATCGCGCTCATCACCAT  ATGCTGTTCTGCCAGCCCTGCTTGTT |
| FoxC1 | CAGCAGAACTTCCACTCGGT  AGAGACTGGCTGGAAGGGAA |
| Fli1 | AGCGTTAGCAAATGCAGCAAGCTGGT  ATTGCCTCACATGCTCCTGTGTCCA |
| GATA-2 | GGGCTAGGGAACAGATCGACG  GCAGCAGTCAGGTGCGGAGG |
| ERG | AACCATCTCCTTCCACAGTGCCCAAA  TTTGCAAGGCGGCTACTTGTTGGT |
| TAL-1 | ACCACCAACAATCGAGTGAAGAGGAGAC  CTGTTGGTGAAGATACGCCGCACAA |
| KDR | CCTCTACTCCAGTAAACCTGATTGGG  TGTTCCCAGCATTTCACACTATGG |
| NRP1 | GGGGCTCTCACAAGACCTTC  GATCCTGAATGGGTCCCGTC |
| CD34 | TGGACCGCGCTTTGCT  CCCTGGGTAGGTAACTCTGGG |
| CD133 | AAACATTCACCAGCAACGAG  ACCAAAACAAATTCAAGGGGTC |
| CD31 | GAGTCCTGCTGACCCTTCTG  ATTTTGCACCGTCCAGTCC |
| VE-cadherin | GCAGCAGCAGGTGCTAACC  TTGCCCACATATTCTCCTTTG |
| VWF | CCCGAAAGGCCAGGTGTA  AGCAAGCTTCCGGGGACT |
| ETV2 | GTACAAGCTCATCCCTGGCA  CACGGAAGAGCCTGAGAGTC |
| β-actin | GCTACGTCGCCCTGGACTTC  GTCATAGTCCGCCTAGAAGC |

**Table S2. Antibodies used for immunofluorescence and FACS analysis.**

| Antibody | Source | Catalog # | Purpose | Host Species & Reactivity | Concentration |
| --- | --- | --- | --- | --- | --- |
| AcLDL | Invitrogen | L-3484 | IF | ECs | 10 μg/ml |
| CD31 | Abcam | ab199012 | IF | Mouse anti-human | 1:200 |
| CD31 | Abcam | ab28364 | IF | Rabbitanti-human/mouse | 1:200 |
| ETV2 | Abcam | ab181847 | IF | Rabbitanti-human | 1:200 |
| vWF | Abcam | ab154193 | IF | Rabbitanti-human | 1:150 |
| TEK | Abcam | ab24859 | IF | Mouse anti-human | 1:200 |
| CD34 | Abcam | ab198395 | IF | Rabbitanti-human | 1:100 |
| VE-cadherin (CD144) | Abcam | ab33168 | IF | Rabbitanti-human | 1:200 |
| NRP1 (Neuropilin-1) | Abcam | ab81321 | IF | Rabbitanti-human | 1:200 |
| KDR (CD309) | Abcam | ab194806 | IF | Rabbitanti-human | 1:200 |
| a-SMA | Proteintech | 14395-1-AP | IF | Rabbitanti-human | 1:150 |
| dylight 488 conjugated IgG | Bethyl | A23220 | IF | donkey anti-rabbit | 1:100 |
| dylight 594 conjugated IgG | Bethyl | A23440 | IF | goat anti-rabbit | 1:100 |
| CD31-PE/Cy7 | Biolegend | 303118 | FC | Mouse anti-human | 1:100 |
| CD34-PE | Biolegend | 343506 | FC | Mouse anti-human | 1:100 |
| KDR-APC | Biolegend | 359915 | FC | Mouse anti-human | 1:100 |
| NRP1-APC | Biolegend | 354506 | FC | Mouse anti-human | 1:100 |
| TEK-PE | Biolegend | 334206 | FC | Mouse anti-human | 1:100 |
| CD144-APC | Biolegend | 348507 | FC | Mouse anti-human | 1:100 |
| CD29-PE | Biolegend | 303004 | FC | Mouse anti-human | 1:100 |
| CD44-PE | Biolegend | 303118 | FC | Mouse anti-human | 1:100 |
| CD90-PE | Biolegend | 328110 | FC | Mouse anti-human | 1:100 |
| CD105-PE | Biolegend | 323206 | FC | Mouse anti-human | 1:100 |
| CD45-PE | Biolegend | 304008 | FC | Mouse anti-human | 1:100 |
| HLA-DR-PE | Biolegend | 327008 | FC | Mouse anti-human | 1:100 |
| IgG-PE/  Cy7 | Biolegend | 400125 | FC | Mouse IgG1, κ Isotype Control | 1:100 |
| IgG-PE | Biolegend | 400112 | FC | Mouse IgG1, κ Isotype Control | 1:100 |
| IgG-PE | Biolegend | 400312 | FC | Mouse IgG2b, κ Isotype Control | 1:100 |
| IgG-APC | Biolegend | 400220 | FC | Mouse IgG2a, κ  Isotype Control | 1:100 |
| IgG-APC | Biolegend | 400119 | FC | Mouse IgG1, κ Isotype Control | 1:100 |
