# Supplementary material for: Conversion of human adipose-derived stem cells into functional and expandable endothelial-like cells for cell-based therapies
Source: Stem Cell Res Ther. 2018 Dec 17;9:350. doi: 10.1186/s13287-018-1088-6 (PMC6296081; doi:10.1186/s13287-018-1088-6)
Supplement: Supplementary file 2 — Figure S1. Characterization of hADSCs. Figure S2. Characterization of ETV2-hADSCs. Figure S3. ECs were generated from ETV2-hADSCs did not through pluripotent and early mesoderm stage. Figure S4. Investigation of signaling molecules of important pathway in regulating EC-fate conversion. Figure S5. Isolation and characterization of KDR−- cells. Figure S6. Downregulation of ETV2 promotes the maturation of KDR+ cells. Figure S7. Long-term blood recovery and tumorigenic assessment of mature EiECs in vivo. (DOCX 18378 kb) [file 13287_2018_1088_MOESM2_ESM.docx]

**Supplemental figures**

**Figure S1**

**
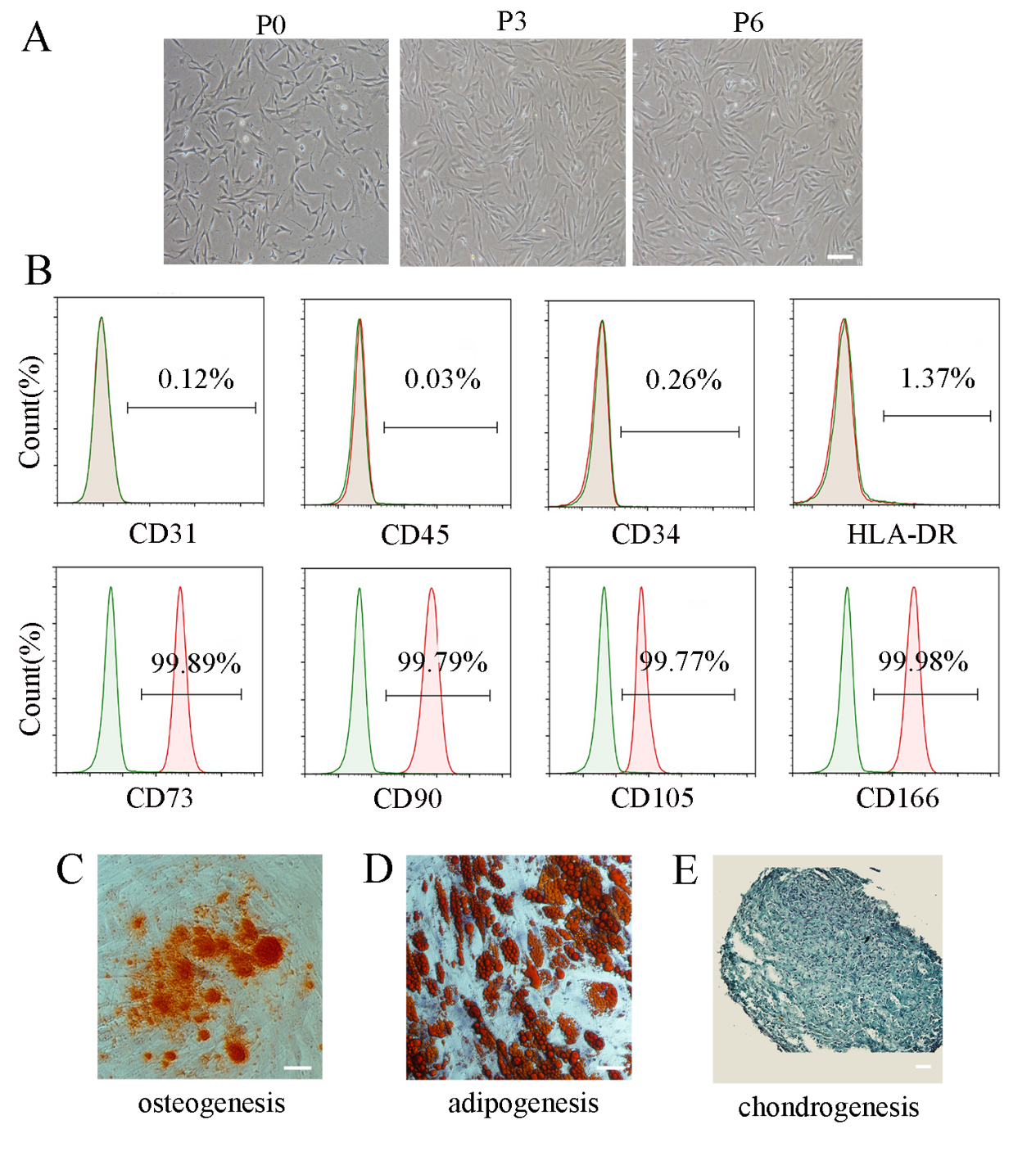
**

**Figure S1 Characterization of hADSCs**

(A) Representative bright-field micrographs of hADSCs at different passages. (B) Flow-cytometric analysis of cell surface antigens of untransduced hADSCs. (C-E) Lineages differentiation of ETV2-hADSCs into adipo-, osteo-, and chondrogenic cells were tested. Scale bar =50μm.

**Figure S2**


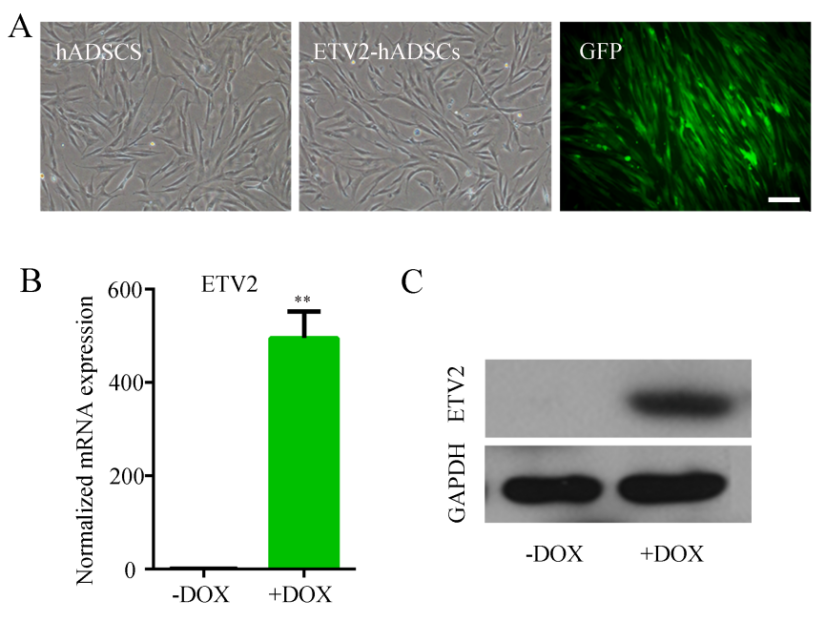


**Figure S2.** **Characterization of ETV2-hADSCs**

(A) Representative bright-field micrographs of hADSCs and ETV2-hADSCs. ETV2-hADSCs were isolated based on the expression of GFP. (B-C) DOX-inducted expression of ETV2 was measured by quantitative RT-PCR (B) and Western Blotting (C). Scale bar =50μm. **P* < 0.05, ***P* < 0.01.

**Figure S3**


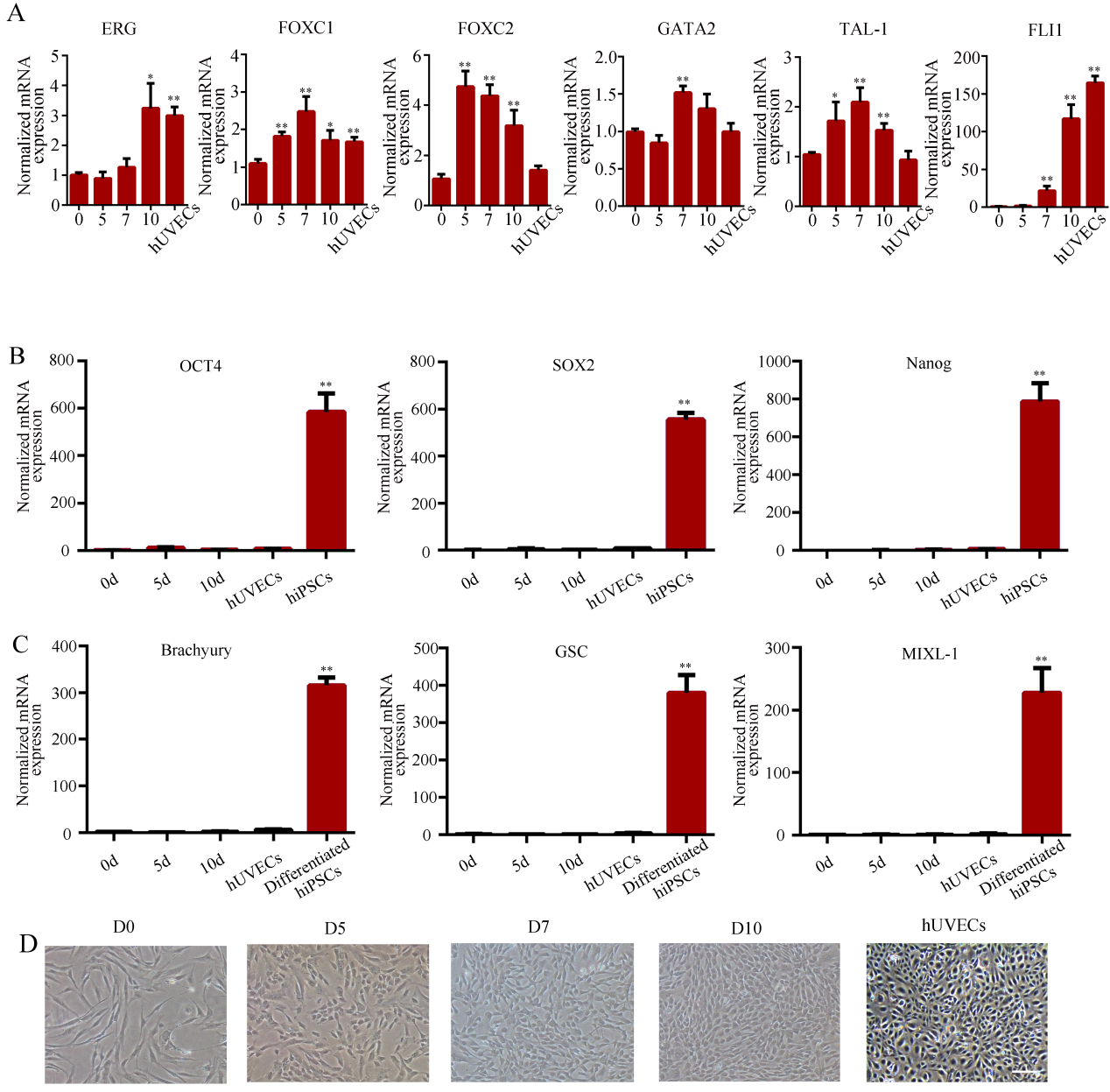


**Figure S3. Conversion of ETV2-hADSCs into ECs did not via the pluripotent and early mesoderm states**

1. Induced ETV2-hADSCs exhibited cobblestone appearance during EC induction. (B) Endothelial development genes were detected by quantitative RT-PCR in ETV2-hADSCs at the indicated time points. (C) Pluripotency genes were detected by quantitative RT-PCR in ETV2-hADSCs during endothelial induction, hiPSCs served as positive control. (D) The early mesodermal genes were detected by quantitative RT-PCR during endothelial induction, differentiated hiPSCs were used as positive control. Data are normalized to β-actin. **P* < 0.05, ***P* < 0.01.

**Figure S4**


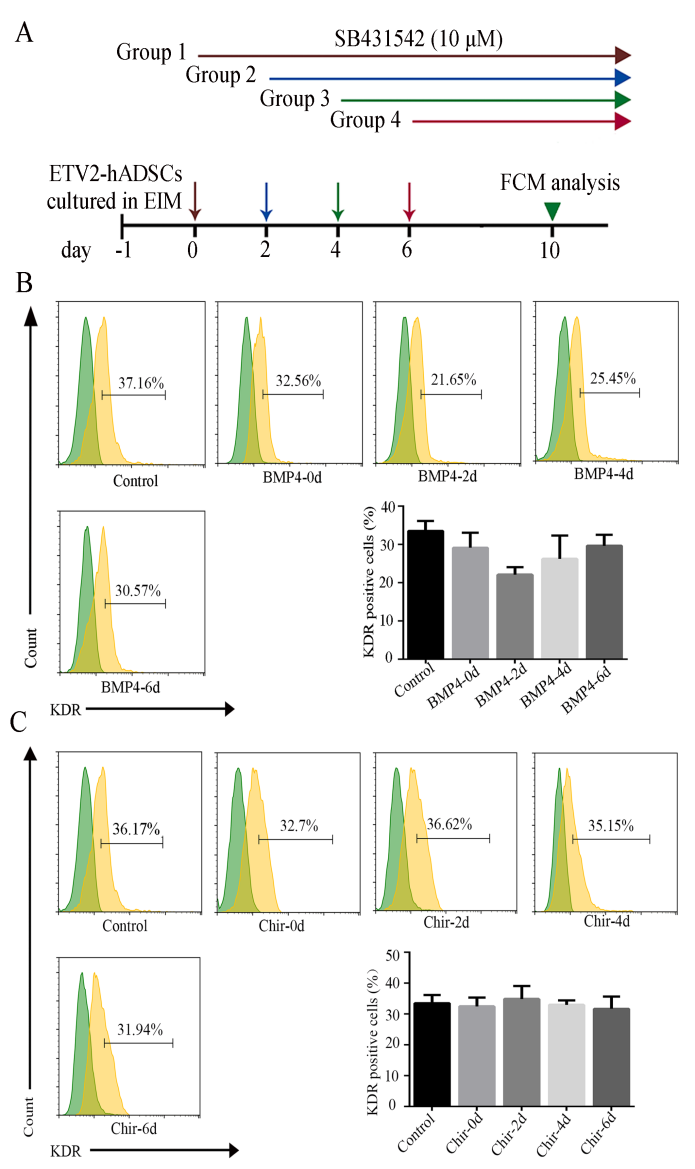


**Figure S4**. **Investigation of signaling molecules of important pathway in regulating EC-fate conversion**

(A) Taking SB treatment as an example, the schematic of the reagent treatment protocol. (B) Representative flow cytometric analysis of KDR+ cells in ETV2-hADSCs that treated with BMP4 at indicated time points. (C) Representative flow cytometric analysis of KDR+ cells in ETV2-hADSCs that treated with Chir99021 (Chir) at indicated time points. Cells cultured in EIM were treated as control in each group.

**Figure S5**


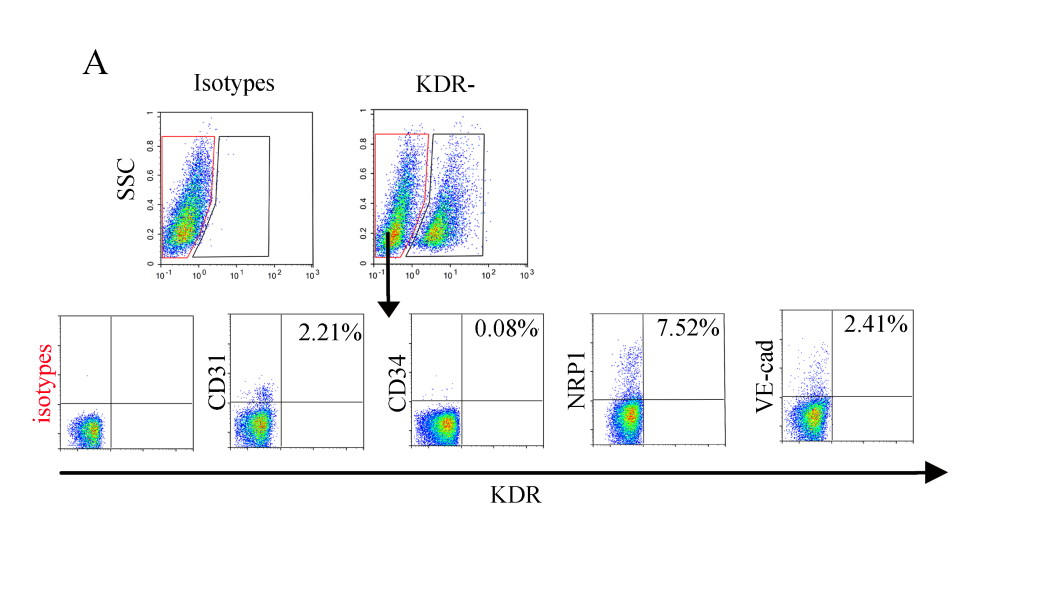


**Figure S5**. **Isolation and characterization of KDR- cells**

KDR- cells were enriched by flow cytometry on day 10 post induction, and phenotypic analysis of KDR- cells were conducted.

**Figure S6**


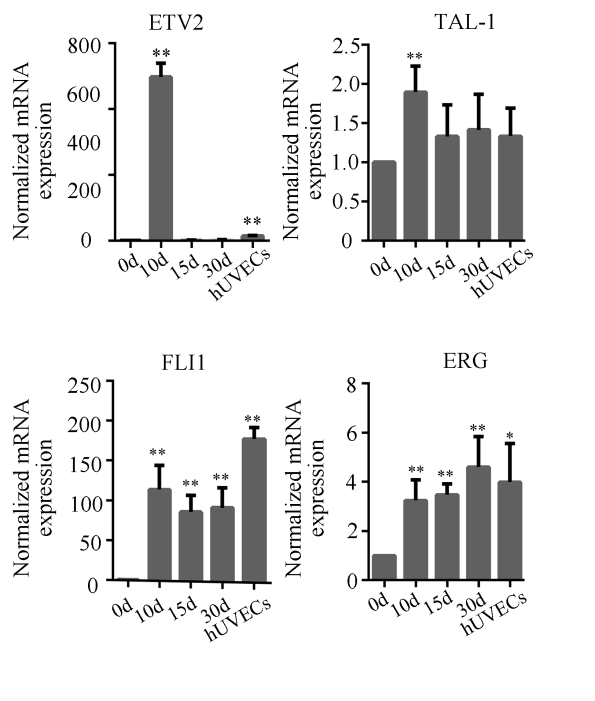


**Figure S6. Downregulation of ETV2 promote the maturation of KDR+ cells**

Sorted KDR+ cells cultured in endothelial maintenance medium (EMM) with or without DOX, EC-specific ETS family transcription factors were detected by quantitative RT-PCR at different time points during endothelial maturation. quantitative RT-PCR data are normalized to β-actin. **P* < 0.05, ***P* < 0.01.

**Figure S7**


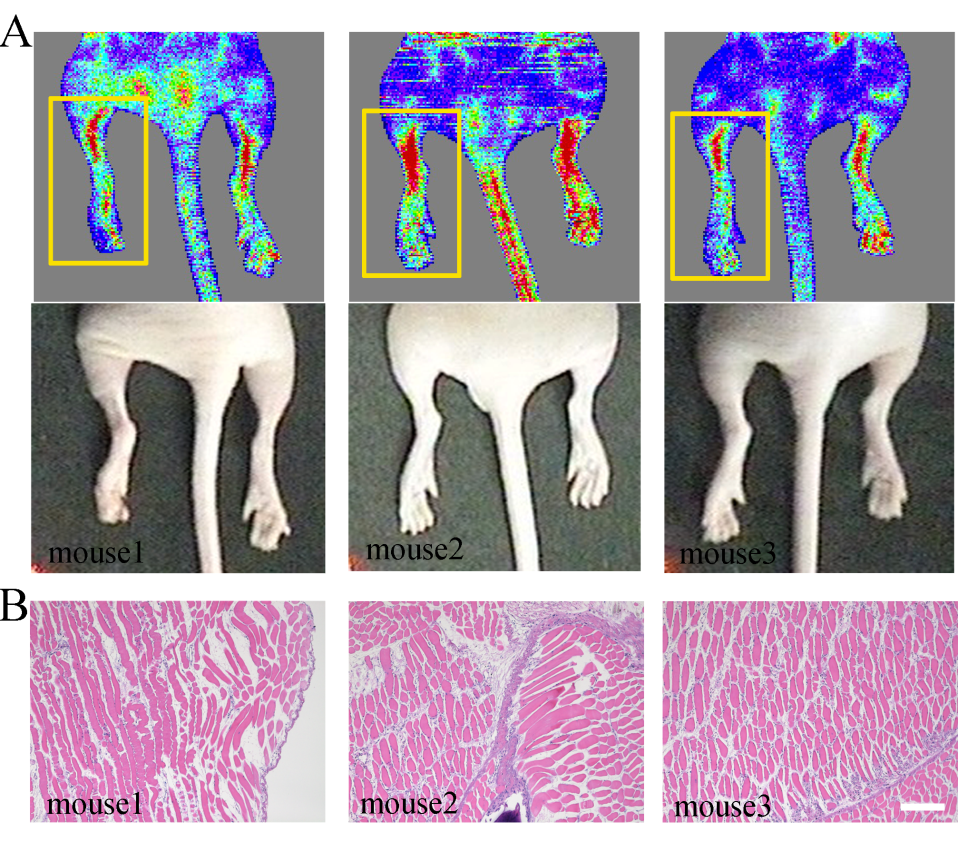


**Figure S7. Long-term blood recovery and tumorigenic assessment of mature EiECs *in vivo***

(A) Representative Doppler images of blood flow in lower limbs of experimental mice. (B) Hematoxylin and Eosin (HE) staining of sections showed no tumor formation in adductor muscle from random sample of mice implanted with mature EiECs for 4 months. Scale bar =50μm.
